# Influence of Sulfonamide Contamination Derived from Veterinary Antibiotics on Plant Growth and Development

**DOI:** 10.3390/antibiotics9080456

**Published:** 2020-07-28

**Authors:** Mi Sun Cheong, Kyung Hye Seo, Hadjer Chohra, Young Eun Yoon, Hyeonji Choe, Vimalraj Kantharaj, Yong Bok Lee

**Affiliations:** 1 Division of Applied Life Science (BK 21 Plus Program), Gyeongsang National University, Jinju 52828, Korea; mscheong@gnu.ac.kr (M.S.C.); chohra.hadjer@gmail.com (H.C.); yye209@gmail.com (Y.E.Y.); mulberry1028@naver.com (H.C.); vimalrajk25@gmail.com (V.K.); 2 Institute of Agriculture and Life Science (IALS), Gyeongsang National University, Jinju 52828, Korea; 3 Department of Herbal Crop Research, National Institute of Horticultural and Herbal Science, RDA, Eumsung 27709, Korea; seokh@korea.kr

**Keywords:** folate, veterinary antibiotics, sulfonamide, plant growth

## Abstract

Veterinary antibiotics such as sulfonamides are widely used to increase feed efficiency and to protect against disease in livestock production. The sulfonamide antimicrobial mechanism involves the blocking of folate biosynthesis by inhibiting bacterial dihydropteroate synthase (DHPS) activity competitively. Interestingly, most treatment antibiotics can be released into the environment via manure and result in significant diffuse pollution in the environment. However, the physiological effects of sulfonamide during plant growth and development remain elusive because the plant response is dependent on folate biosynthesis and the concentration of antibiotics. Here, we present a chemical interaction docking model between Napa cabbage (*Brassica campestris*) DHPS and sulfamethoxazole and sulfamethazine, which are the most abundant sulfonamides detected in the environment. Furthermore, seedling growth inhibition was observed in lentil bean (*Lens culinaris*), rice (*Oryza sativa*), and Napa cabbage plants upon sulfonamide exposure. The results revealed that sulfonamide antibiotics target plant DHPS in a module similar to bacterial DHPS and affect early growth and the development of crop seedlings. Taking these results together, we suggest that sulfonamides act as pollutants in crop fields.

## 1. Introduction

Antibiotics are biologically active molecules used to treat or control various bacterial, protozoal, and fungal infections [1,2]. The use of antibiotics as antimicrobial agents in both humans and animals has increased worldwide due to the need for therapeutic treatments for infections and diseases caused by bacteria [3,4]. Sulfonamides are the oldest chemically synthesized antibiotic molecules, first developed in the 1930s, with more than 5000 derivatives commonly used around the world [5]. Most sulfonamide antibiotics employed in both human and veterinary medicine remain bioactive as a metabolite, even after being excreted from the treated individual’s body [6]. The annual usage of sulfonamide drugs in animal husbandry or veterinary medicine is estimated to represent approximately 10–23% of total antibiotic use in some EU countries and South Korea due to their broad-spectrum antimicrobial activity [7,8]. Moreover, the use of antibiotics for veterinary purposes is approximately five times higher than that for humans [3].

**Figure A1:**
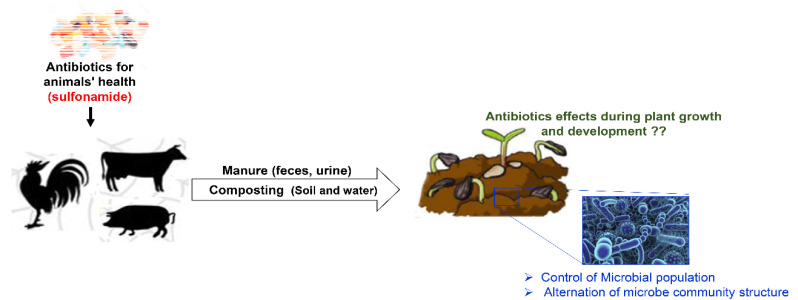
The flow of sulfonamides into the soil environment. Veterinary antibiotics, including sulfonamides, are antimicrobial agents used for animal health and production. Most veterinary antibiotics are excreted with manure due to poor absorption in animal intestines and are released into the soil environment. Contaminated soils affect microbial growth and community structure (depending on antibiotic-resistant traits) and may affect plant growth and development.

However, as much as 90% of antibiotic intake is excreted in feces and manure because antibiotics are poorly absorbed by the gut [9,10,11]. These substances are then released into the agricultural environment by the direct application of organic manure for soil fertilization [6]. As such, the extensive application of veterinary antibiotics has resulted in the frequent and ubiquitous detection of antibiotics, including sulfonamides, in the environment [5,6]. Subsequently, these contaminants are accumulated in the soil and affect soil microorganisms such as community structures and populations that depend on antibiotic resistance (Figure A1) [12].

As plants are immobile organisms, crop plants change their growth and development to respond to the external environments and further affect their yield and quality [13,14]. As antibiotics are an environmental stress factor, plant growth and development can be altered. Unfortunately, it remains elusive how antibiotics in the soil have biological and physiological effects on plant growth and development. Antibiotics released into the soil are taken up by plants, where they can contaminate food crops and threaten human health [15,16]. Therefore, it is important to understand the potential impacts of antibiotics on plant growth and development.

**Figure 1:**
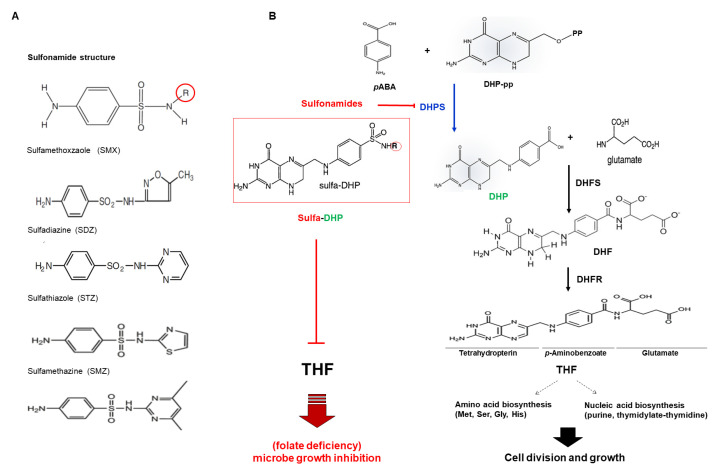
Folate biosynthesis inhibition by sulfonamide. (**A**) Sulfonamide structures: sulfamethoxazole (SMX), sulfadiazine (SDZ), sulfathiazole (STZ), sulfamethazine (SMZ). (**B**) Tetrahydrofolate (THF) biosynthesis inhibition by sulfonamide. Pyrophosphorylated DHP (DHP-pp) is conjugated with *p*ABA by DHPS, subsequently coupled with glutamate, and reduced by dihydrofolate synthetase (DHFS) and dihydrofolate reductase (DHFR), respectively. Tetrahydrofolate (THF) is newly synthesized as the basic three structural moieties for folate; tetrahydropterin, *p*-Aminobenzoate, and glutamate. THF and its derivatives are collectively termed folates. Folate plays a role in C1 transfer reactions such as amino acid biosynthesis and nucleic acid biosynthesis biochemically, and it influences cell growth and development biologically. Sulfonamide possess a similar structure to *p*ABA, reacts with DHP-pp, and forms sulfa-DHP by DHPS. Sulfa-DHP inhibits further steps, causing folate deficiency and microbe growth inhibition. *p*ABA, para-aminobenzoic acid; DHP-pp, dihydropteroate pyrophosphate; DHF, dihydrofolate; DHP, dihydropteroate.

Interestingly, the mode of action (MOA) of sulfonamides is well defined for antimicrobial activity, which targets and interferes with the folate biosynthetic pathway and inhibits the growth of microorganisms [5,15] (Figure 1).

Like bacteria, plants also have a folate biosynthetic pathway [16], which plays important roles in plant growth development, allowing us to speculate that sulfonamide antibiotics affect plant growth and development by interfering with folate biosynthesis. In this study, we evaluate whether sulfonamide drugs (1) act as a molecular inhibitor of dihydropteroate synthetase (DHPS) in the folate biosynthesis pathway in plants through molecular docking analysis using a Napa cabbage (*Brassica campestris*) enzyme and (2) inhibit plant growth and development by performing a seedling growth assay with crop plants lentil bean (*Lens culinaris*), rice (*Oryza sativa*), and Napa cabbage (*Brassica campestris*).

### 1.1. Para-Aminobenzoic Acid (*p*ABA) as an Endogenous Analog of Sulfonamide

Efforts to identify the targets of antibiotics have led researchers to explore metabolic pathways, for example, through computational analysis trials, which have shown that many members of a pathway bind to structurally similar endogenous substrates and products [17,18]. The basic sulfonamide structure includes the sulfonamide group and the amino group in the para position of the benzene ring. Sulfonamide derivatives are obtained by substitution of the R structure of nitrogen in the sulfonamide group (Figure 1A).

*p*ABA (*para*-aminobenzoic acid), which consists of a benzene ring substituted with amino and carboxyl groups, is structurally similar to sulfonamide antibiotics (Figure 1A). *p*ABA is biochemically involved in a wide variety of metabolic processes and possesses antioxidant, anti-mutagenic, protective, and reparative properties (Figure 1B) [19,20].

As predicted, the sulfonamides were able to substitute for *p*ABA in folate biosynthesis and act as alternative substrates to form sulfa-DHP by DHPS (Figure 1B), indicating that sulfonamides play a role as competitive inhibitors of DHPS. In addition, the *Saccharomyces cerevisiae* DHPS-knockout strain shows sulfonamide-insensitive cells [21], suggesting that sulfonamides directly attack the DHPS enzyme. At the sequential step for folate biosynthesis, sulfa-DHP could not react with dihydrofolate synthetase (DHFS), stopping the downstream pathway for folate biosynthesis (Figure 1). Hence, sulfonamide drugs can result in folate deficiency and influence cell division and growth.

### 1.2. Folate in Plants

Folates are soluble vitamins that mediate the transfer of one-carbon (C1) units in a set of reactions, which is commonly referred to as C1 metabolism [16,22]. The C1 transfer reaction of folate metabolism plays a crucial role in all living organisms as it involves nucleic acids, proteins, lipids, and other biomolecules, as well as epigenetic controls [16,22,23]. Therefore, inhibiting folate biosynthesis affects the growth and development of living organisms, such as microorganisms and plants, which have a folate biosynthesis cycle. Notably, mammals require a dietary supply of soluble vitamins due to a lack of folate biosynthesis.

**Figure 2:**
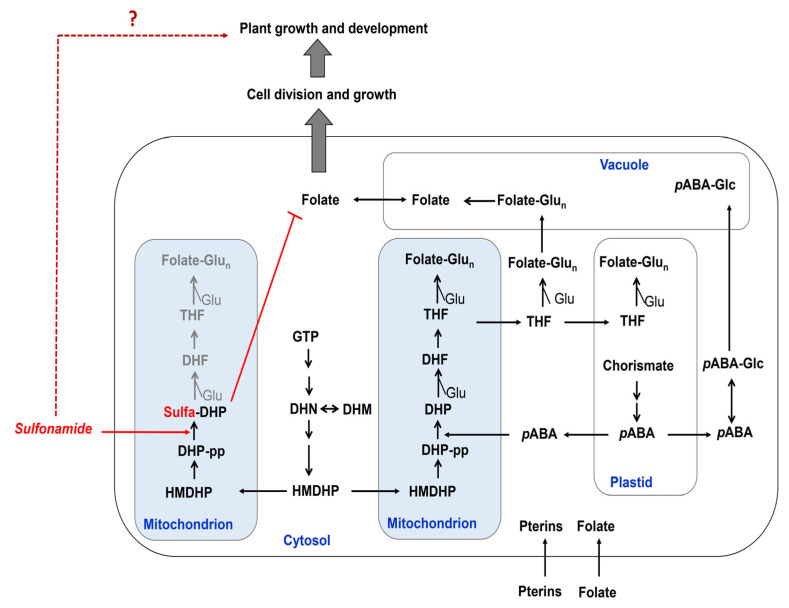
The folate biosynthetic pathway in a plant cell. Pterin synthesis starts with GTP conversion into HMDHP by several steps in the cytosol. *p*ABA synthesis occurs in plastids from a chorismate precursor. All three structural parts (pterin, *p*ABA, and glutamate) are assembled in the mitochondria and form THF by sequential steps. Sulfonamides can replace *p*ABA and convert sulfa-DHP, a newly synthesized metabolite in a plant cell system. Sulfa-DHP terminates the other subsequent steps for folate biosynthesis. GTP, guanosine triphosphate; HMDHP, 6-hydroxymethyldihydropterin; DHN, dihydroneopterin; DHM, dihydromonapterin; HMDHP DHF, dihydrofolate; DHP, dihydropteroate; DHP-pp, dihydropteroate pyrophosphate; *p*ABA, para-aminobenzoic acid; THF, tetrahydrofolate; Glu, Glutamate; THF-Glu(n), tetrahydrofolate polyglutamate; Glc, glucose.

Plant folate biosynthesis requires complex subcellular compartmentation. Folates consist of three distinct chemical moieties linked together: a pterin, a *p*-aminobenzoic acid (*p*ABA), and a glutamate. The biosynthesis and assembly of these individual structural parts are compartmentalized in plant cells: plastids, cytosols, vacuoles, and mitochondria (Figure 2). The pterin ring moiety from guanosine triphosphate (GTP) in the cytosol and *p*ABA formed from chorismate in plastids are then glutamylated and reduced in mitochondria (Figure 2) [18,23,24,25]. More specifically, *p*ABA and 6-hydroxymethyldihydropterin (HMDHP) are targeted in mitochondria through simple diffusion-mediated translocation and assembled by mitochondrial enzymes (Figure 2) [26]. As a first step of assembly of the three moieties during folate biosynthesis, 7,8-dihydropteroate (DHP) was detected only in mitochondria [26], indicating that this catalyzing enzyme is located in mitochondria. Molecular and biochemical experiments showed that bifunctional HPPK-DHPS (EC 2.5.1.15) catalyzes this reaction and builds an identical oligomeric structure in the matrix [26].

Initially, 6-hydroxymethyl-7,8-dihydropterin (HMDHP) converts into 6-hydrozymethyl-7,8-dihydropterin pyrophosphate (HMDHP-pp) through the action of the HPPK (HMDHP pyrophosphokinase) domain (Figure 2). The DHPS domain subsequently catalyzes the condensation of HMDHP-pp with *p*ABA to yield 7,8-dihydropteroate (Figure 1B and Figure 2). Then, a DHFS (EC 6.3.2.12), a DHFR (EC 1.5.1.3), and an FPGS (folylpolyglutamate synthetase, EC 6.3.2.17) function sequentially (Figure 1B and Figure 2). The pterin ring of folate exists naturally in dihydro or tetrahydro form, and the ring is fully oxidized in folic acid. Tetrahydrofolate (THF) and its derivatives are collectively termed folates. Furthermore, sulfonamides can be converted to sulfa-DHP by DHPS in mitochondria (Figure 1B and Figure 2) [27,28], which acts as a competitor of *p*ABA. Sulfonamides inhibit plant DHPS by decreasing the chance to react with *p*ABA, blocking subsequent steps for folate biosynthesis, and they may influence the accumulation of the folate level, suggesting that sulfonamides cause folate deficiency in plants and further influence plant growth and development (Figure 2).

## 2. Results

### 2.1. Plant Dihydropteroate Synthase (DHPS)

The crystal structures of bacterial-type DHPS from *Bacillus anthracis* [28] and *Escherichia coli* [29] have been thoroughly documented; however, the crystal structure of plant DHPS has not yet been reported experimentally. The enzyme dihydropteroate synthase (DHPS; EC 2.5.1.15) in *E. coli* is a dimeric triosephosphate isomerase; the single domain of monofunctional DHPS binds 7,8-dihydropterin pyrophosphate in deep clefts and sulfonamides bind it closer to the surface [29].

**Figure 3:**
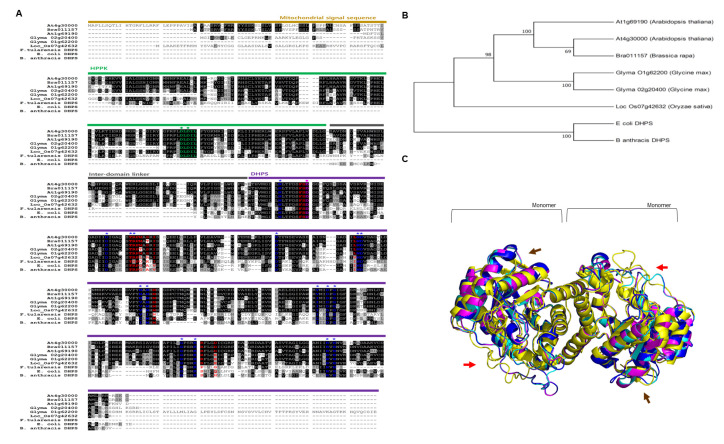
The structural relevance of DHPS enzymes from different species. (**A**) The primary structure of the DHPS enzyme. The DHPS sequences were retrieved from GenBank; At (*Arabidopsis thaliana*), Bra (*Brassica campestris*), Glyma (*Glycine max*), Loc_Os (*Oryza sativa*), *F. tularensis*, *E. coli*, and *B. anthracis* using Clustal W. Blue and red residues are substrate-binding and sulfonamide-resistance residues, respectively. Strictly conserved amino acids are black, and similar residues are gray. Yellow: Mitochondrial signal sequence, green: HPPK domain, gray: inter-domain linker, purple: DHPS domain. (**B**) The phylogenetic relationship of bacterial DHPS (*E. coli* and *B. anthracis*) and plant DHPS (*Arabidopsis thaliana*, *Brassica campestris*, *Glycine max*, and *Oryza sativa*). All deduced amino acid sequences were compared within a phylogenetic tree by a neighbor joining method (Mega7). (**C**) The structural superposition of EcDHPS (PDB code 5U10, yellow), Bra011157 (cyan), Loc_Os07g42632 (blue), and Glyma 02g20400 (magenta).

To determine the plant DHPS in this study, we obtained the sequence information of plant DHPS genes for *Brassica campestris* (Bra011157) and two homologous genes from *Arabidopsis thaliana* (At4g30000 and At1g69190), *Oryza sativa* (Loc_Os07g 42632), and *Glycine max* (Glyma 02g20400 and Glyma 01g62200) using a plant genomic resource (Phytozome12; https://phytozome.jgi.doe.gov/pz/portal.html), the Brassica database BRAD (http://brassicadb.org/brad/), and the rice database (http://rice.plantbiology.msu.edu/). Using previous reports of bacterial DHPS, including those of *B. anthracis* [28], *E. coli* [29], and *F. tularensis* [30], we compared and analyzed the primary structure of DHPS of all these bacteria and plants using Clustal W (Figure 3A). As DHPS plays a role in the mitochondrial matrix, plant DHPSs possess *N*-terminal mitochondrial signal sequences (shown in yellow). As the bifunctional enzyme types for HPPK-DHPS, HPPK domains and DHPS domains are clearly distinguished in green and purple, respectively (Figure 3A). With the deduced amino acid sequence, we used black to represent the identical residues and gray for similar residues, then further compared all of these primary structures using a phylogenetic tree developed by a neighbor joining method (Figure 3B). The result indicates that plant DHPSs, as HPPK-DHPS bifunctional types, are classed as a different group from monofunctional bacterial DHPS. However, the HPPK domain has conserved catalytic residues DLDIL for pyrokinase activity (◆, green) as a bifunctional type of DHPS in plants. In addition, not only the Asp (D) residues (◆, pink) from the catalytic core of DHPS but also substrate-binding residues (*, blue) are identical in all the DHPS domains. Interestingly, the residues that give sulfonamide resistance [30] are also conserved (red; Figure 3A).

Next, we further compared the primary structure of DHPS using three representative genes: *B. campestris* HPPK-DHPS (BcHPPK-DHPS), *F. tularensis* HPPK-DHPS (FtHPPK-DHPS), and *E. coli* DHPS (EcDHPS). The results indicate that BcHPPK-DHPS (Bra011157) is a functional ortholog exhibiting high similarity and identity; 37.9% identity and 58.9% similarity were observed between EcDHPS and FtHPPK-DHPS, which are both catalytically active enzymes in in vivo and in vitro experiments [28,29]. Theoretically, BcHPPK-DHPS exhibits 43.9% identity and 65.6% similarity with EcDHPS [30,31] and 37.02% identity and 61.4% similarity with FtHPPK-DHPS. These results suggest that the bifunctional HPPK-DHPS enzymes, including *B. campestris* DHPS (Bra 011157), are active enzymes with very similar three-dimensional (3D) structure. In addition, bifunctional HPPK-DHPS is likely a genetically evolved form of the monofunctional DHPS enzyme because two sequential enzymatic reactions in a single polypeptide can be more substrate-specific and more rapid by not detecting intermediate products.

Given that sulfa-DHP is synthesized by sulfonamides serving as an alternative substrate of DHPS (Figure 1B and Figure 2), sulfa-DHP structurally presents as an analog of dihydropteroate (DHP). However, the folate biosynthesis pathway is terminated as sulfa-DHP cannot be a substrate of DHFS, which produces DHF by conjugating both DHP and glutamate, indicating that the level of sulfa-DHP is crucial for determining the degree and nature of folate biosynthesis [29,32] and further suggesting that sulfonamides inhibit microbe growth via the activity of target DHPS [21]. We hypothesized that the catalytic process of plant DHPSs with sulfonamide influences plant folate biosynthesis and subsequently results in altered plant growth and development. Before accessing the interaction between plant DHPSs and sulfonamide, we initially generated superposition states and compared the 3D structures (Figure 3C) of *E. coli* DHPS (EcDHPS, yellow), BcDHPS (Bra 011157, cyan), OsDHPS (Loc_Os07gg42632, blue), and GmDHPS1 (Glyma 02g20400, Magenta). Based on the established EcDHPS structure (yellow, PDB code 5U10), the results indicate that the other three plant DHPS proteins are overlapped (Figure 3C). A stereoview of a dimerized form with ribbon diagrams shows that the helix and sheets of fragmentally aligned structures lying on a catalytic cleft overlap almost exactly in the internal regions (Figure 3C). In addition, although the amorphous features of the surface structures (red and brown arrows) are slightly different, the twisted torsion of a stereo-superposition is very minor. The red arrow positions are S300 to S332 for BcDHPS, A295 to A327 for OsDHPS, A310 to S342 for GmDHPS1, and V71 to P100 for EcDHPS. The brown arrows represent I441 to A456 for BcDHPS, I452 to V466 for OsDHPS, I436 to A449 for GmDHPS1, and F207 to L211 for EcDHPS (Figure 3A,C). These data suggest that all four proteins have the same function in the biochemical enzyme reaction.

### 2.2. DHPS as a Sulfonamide Target

**Figure 4:**
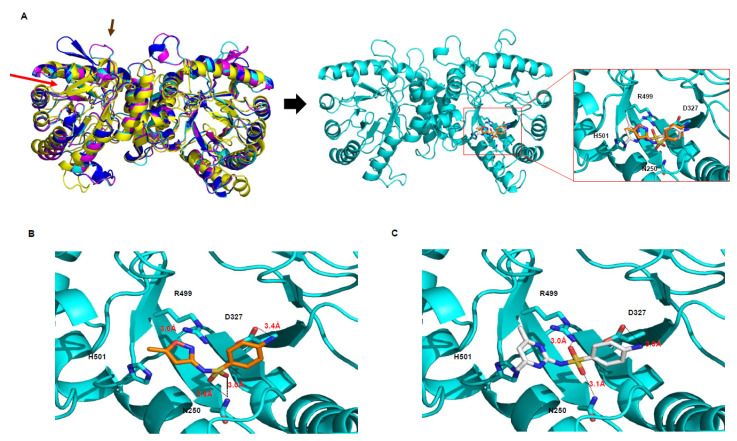
A docking model between sulfonamide and DHPS. (**A**) The dimeric form of DHPS rotated 90°. The target DHPS was designated from BcDHPS (Bra011157; cyan) for docking sulfonamides, and the red boxed region shows a zoomed-in view of only the active site for reaction with both sulfamethoxazole (orange) and sulfamethazine (gray). Red and brown arrows are the same as in Figure 3C. Docking view of sulfamethoxazole (orange, (**B**)) and sulfamethazine (gray, (**C**)) at the active site. The side chains of Asp 327, Arg499, His272, and Asn250 are close to nitrogen and oxygen on the sulfonamide ring. Cyan depicts an active site feature of the BcDHPS enzyme.

To understand the catalytic reaction of plant DHPSs with sulfonamide, we simulated a docking model between the BcDHPS enzyme and sulfonamides, both sulfamethoxazole (SMX) and sulfamethazine (SMZ), which are the most detected derivatives in the environment (Figure 4). The molecular docking analysis using AUTODOCK and Phyre2 software shows that these drugs bind at the catalytic cleft of BcDHPS (cyan, Figure 4A), on which the *p*ABA substrate is placed. The zoomed-in view shows the active site for the reaction between *p*ABA and sulfonamide (red box, Figure 4A).

According to the stereochemistry, the amine group of the benzene ring is positioned close to Asp327, where it forms a hydrogen bond between the blue nitrogen (*N*) and red oxygen (*O*) of Asp, shown by black dashed lines (Figure 4B,C). The distance is 3.4 Å for SMX (Figure 4B) and 3.9 Å for SMZ (Figure 4C). Notably, the nitrogen at the other ring—denoted the *R* group in Figure 1A—of both sulfonamides faces backward due to the nitrogen (*N*) of His501, and this position contributes to determining the distance from Arg499 to the ring of sulfonamides in both SXZ and SMZ. Furthermore, two oxygens (*O*) form two wings from the sulfur center and are fitted in the cleft by hydrogen bonds with the nitrogen (*N*) of Asn 250 and/or Arg499. Based on AUTODOCK analysis, the extended docking simulation of sulfonamides into BcDHPS shows that SMX and SMZ molecules are clustered with average binding energy values (ΔG) of −6.56 kcal/mol and −6.95 kcal/mol, respectively, which were calculated using more than 300 individually different docking positions. These analyses showed that SMZ and SMX interact directly with the catalytic residues of the DHPS enzyme, which is a structurally conserved region even in different species, including bacteria and plants [28,29,30]. Collectively, these results suggest that sulfonamides interact with structurally and catalytically conserved residues of BcDHPS, thereby blocking subsequent folate biosynthesis. In addition, we further suggest that sulfonamide antibiotics may exhibit a synergistic impact on folate biosynthesis in plants by blocking sequential and coupled catalytic reaction activity since plant DHPSs are structurally HPPK-DHPS bifunctional enzymes (Figure 3A).

### 2.3. Phytotoxicity of Sulfonamides During Plant Growth and Development

**Figure 5:**
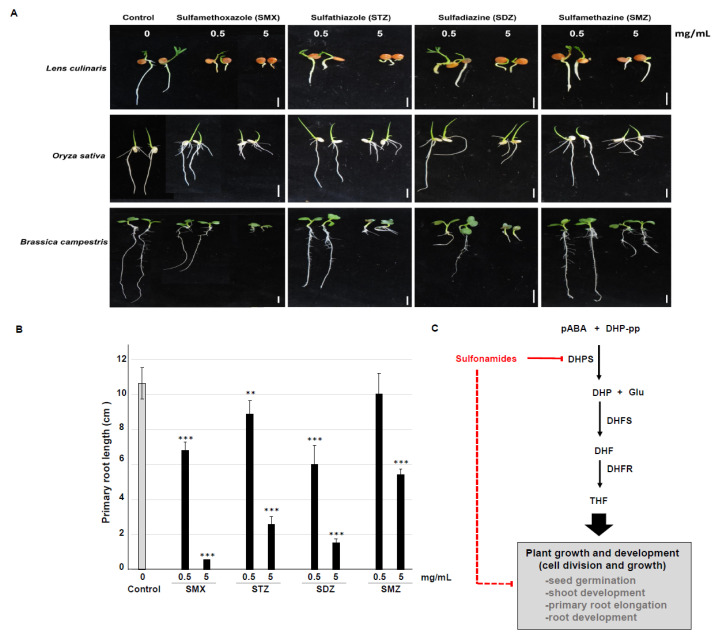
Seedling growth inhibition by sulfonamides and its suggested model. (**A**) Physiological phenotype of seedlings exposed to sulfonamide. Seeds were placed onto 1.2% agar plates containing 0 mg/L, 0.5 mg/L, or 5 mg/L of the indicated sulfonamide and grown vertically in the growth chamber; photos were taken on Day 7. The seeds used were lentils (*Lens culinaris*), rice (*Oryza sativa*), and Napa cabbage (*Brassica campestris*). The four types of embedded sulfonamides were sulfamethoxazole (SMX), sulfathiazole (STZ), sulfadiazine (SDZ), and sulfamethazine (SMZ). White bars = 1 cm. The experiment was repeated three times, with similar results. (**B**) Quantification of primary root length of Napa cabbage (Brassica campestris). Data represent mean ± SE (*n* = 27). Asterisks indicate statistically significant differences to the control (0 mg/mL, grey bar) (Student’s t-test; ** *p* < 0.01, *** *p* < 0.001). (**C**) A plant growth inhibition model by sulfonamides. Sulfonamides inhibit DHPS in the folate biosynthesis pathway and influence plant growth and development, including seed germination, shoot development, primary root elongation, and root development.

Seed germination and root elongation tests are simple, sensitive, and inexpensive environmental bioassays commonly used to evaluate the phytotoxicity of chemicals to plants [33]. As sulfonamide is a catalytic inhibitor of DHPS, we observed the physiological phenotypes upon application of sulfonamides during seed germination and early growth of seedlings using lentils (*Lens culinaris*), rice (*Oryza sativa*), and Napa cabbage (*Brassica campestris*). As expected, all tested sulfonamides—sulfamethoxazole (SMX), sulfathiazole (STZ), sulfadiazine (SDZ), and sulfamethazine (SMZ)—influenced the plant physiology, such as by delaying seed germination, inhibiting cotyledon opening, shortening the primary root length, and enhancing lateral root development under all dosages of sulfonamide (Figure 5A). More specially, we further determined primary root length of Napa cabbage seedlings (Figure 5A) using ImageJ software (Figure 5B) to conduct the seedling growth reduction. As shown Figure 5A,B, primary root length was significantly inhibited by all indicated sulfonamides; SMZ, STZ, SDZ, and SMZ, although a low concentration (0.5 mg/mL) of SMZ was not different from control (0 mg/mL, grey bar). Statistical analysis represents this growth inhibition is significant (student’s t-test; ** *p* < 0.01, *** *p* < 0.001). These results indicate that sulfonamides directly affect plant growth and development and support the hypothesis that antibiotics are an important environmental pollutant (Figure 5C).

Furthermore, to extend our understanding of how plant physiology varies with different sulfonamide drugs and different plant species, including non-crop plants, we collected and summarized other literature reports related to the effect of sulfonamides on plant growth (Table 1).

The most frequent effects of sulfonamides on plant physiology include impacts on seed germination, root growth and development, chlorophyll content, and nutrient-deficient phenotypes, although all reported studies, including this study, were conducted under artificial conditions. At this moment, we have demonstrated at least that sulfonamides affect plant growth and development and influence crop production.

## 3. Discussion

The soil is the most susceptible environment to contamination via animal manure and composting by synthetic veterinary medicines that are designed to prevent and control infectious diseases in animal production (Figure A1). However, our understanding of their implications for plant growth remains limited. Sulfonamide drugs disrupt the folate biosynthesis pathway by competing with *p*ABA in the condensation reaction with DHP-pp, leading to the depletion of folate and the hindered growth of microorganisms [18].

### 3.1. The Comparison Between DHPS Proteins

According to genome analysis, higher plant species contain a single HPPK-DHPS gene [33]. DHPS acts at a crucial convergence point in the folate pathway, catalyzing the condensation of *p*ABA and DHP-pp to form dihydropteroate (Figure 1B). Plant DHPS is considerably longer than typical prokaryotic DHPS, with an *N*-terminus extended region encoding HPPK, which catalyzes pyrophosphorylation of HMDHP and forms HMDHP-pp and mitochondrial localized signal sequences (Figure 3A). Multiple alignments show that all parts are conserved well, particularly in the regions of the catalytic core and substrate binding (Figure 3). The blue letters with asterisks (*) in Figure 3A are generated structurally (Thr, Asn, Asn, Val, Val, Ile, Asp, Phe, Phe, Gly, Lys, Arg), characterizing the pterin-binding pocket of DHPS and recognizing the pterin ring [29,61]. Specifically, sulfonamides as DHPS inhibitors anchor where *p*ABA lies on the outer area of the pterin-binding pocket with a structure mimicking that of *p*ABA [62,63] (Figure 3A and Figure 4).

### 3.2. Folate and DHPS in Plants

As THF serves as a cofactor in one-carbon (C1) transfer reactions during the synthesis of nucleic acids (purine, thymidylate) and amino acids (Gly, Ser, Met, His) (Figure 1), lowered folate levels affect DNA synthesis and amino acid usage and result in the inhibition of microbe cell growth [21] (Figure A1). In plants, transcript analysis has shown that folate biosynthesis genes, including the bifunctional enzyme HPPK-DHPS, are highly expressed in meristems, expanding cotyledons, and developing embryos [64]. Additionally, high folate levels have been detected in embryos and young tissues [65]. The methylation (C1 unit) status of DNA and histone is used in vivo as an epigenetic regulation marker, and folate metabolism is important for plants to control gene expression for growing, developing, and responding to environmental stresses [66,67,68]. In Arabidopsis, several mutant plants including *atdhf-3*, the genes of which are important for determining the folate level or distribution, show influenced plant growth and development, for example, through shortened and twisted roots, abnormal root development, and morphological hypocotyl elongation [69,70], as well as other typical phenotypes of insufficient nutrient supply [71]. Thus, much evidence in the literature supports the hypothesis that it is important to control the level of folate to ensure effective plant growth and development. Sulfonamides cause folate deficiency and have differential folate level of influences in their strength of antimicrobial activity against microorganism species depending on both the production and use of folate [31].

### 3.3. Sulfonamides and Plant Growth Inhibition

The intensive cultivation in agricultural field requires organic fertilizers to increase the nutrient content of soil as well as to improve physiological properties for crop yield and quality. Animal manure is a good source of nutrients supplement [72], but sulfonamides by animal feed are released into the environment and detected in the surface soil through applying manure in the agricultural field [5,6,7,11]. Although some of the phytotoxicity of sulfonamide has been reported in mostly aquatic plants, these are limited in the physiological views of plant growth (Table 1). Furthermore, the understanding on molecular level for showing the phytotoxicity of sulfonamides and physiological and chemical properties in crop plants of sulfonamide derivatives are not well- defined. For example, though many different derivatives of sulfonamides have been detected in the environment, we still do not know the chemical level of phytotoxicity.

In this study, we showed that (1) sulfonamides may target plant DHPS similar to microbial DHPS (Figure 3 and Figure 4) and that (2) sulfonamides inhibit plant growth and development (Figure 5, Table 1). Interestingly, different sulfonamides of the same concentration exhibit different inhibitory effects (Figure 5A,B), suggesting that a different functional group of the chemicals exhibits different inhibition activity as shown different stereochemistry (Figure 4). Collectively, the inhibitory effect during seedling growth was shown in order sulfamethoxazole (SMX) > sulfathiazole (STZ) > sulfadiazine (SDZ) > sulfamethazine (SMZ) (Figure 5A,B).

However, the following questions remain to understand the role of sulfonamides in plant folate biosynthesis and in further affecting plant growth and development: (1) Are folate levels decreased by sulfonamide treatment in plants? (2) Is sulfonamides’ influence plant tissue- and/or organ-specific? Connected with this second question, (3) do the physiological or molecular responses of plants against sulfonamides depend on the folate level? Finally, (4) although sulfonamides affect plant development, do sulfonamides still have an influence during maturation/senescence?

To address the above questions, we need to perform further research related to understanding the folate level in plants, including the characteristic ranges for different tissues, developmental stages, and plant species. Although regulatory mechanisms for determining folate biosynthesis have been organized in plants [16], the currently accumulating evidence is fragmented, with limited applicability to regulate modules between folate biosynthesis and sulfonamide inhibition.

Interestingly, sulfonamides also act as an inhibitor of carbonic anhydrase (EC4.2.1.1), which possesses ubiquitous highly conserved zinc ion (Zn^2+^) binding active sites in all organisms, including animals, human, plants, bacteria, and archaea [73,74,75]. Therefore, sulfonamides may have another molecular mechanism for controlling cell growth; however, their inhibition behavior and role in plant growth and microorganisms have not yet been explored.

## 4. Materials and Methods

### 4.1. Model Building and Refinement with the 3D Structure of DHPS

To refine the docking model between BcDHPS and sulfonamides (SXZ and SMZ), we operated ModRefiner (https://zhanglab.ccmb.med.umich.edu/ModRefiner/)(Ann Arbor, MI, USA) [76] several times to obtain the most accurate structure. For simulations of *Brassica campestris* DHPS (Bra011157), we used the program AUTODOCK 4.2 (San Diego, CA, USA) [77] for docking calculations and Phyre2 (Protein Homology/Analogy Recognition Engine) (San Diego, CA, USA) [78] to predict the protein homology structure of BcDHPS for all three DHPS proteins, i.e., including rice DHPS (Loc_Os07g42632) and bean DHPS (Glyma 02g02400). The input protein data were in FASTA format, and an accurate model was obtained by comparing the given sequences. In addition, the chemical structures of sulfamethazine and sulfamethoxazole were determined using Dundee PRODRG2 Sever (http://www.ccl.net/chemistry/resources/messages/2005/01/17.002-dir/) (San Diego, CA, USA) [79]. AUTODOCKTOOLS software (http://autodock.scripps.edu/resources/adt) (San Diego, CA, USA) was run to generate the docking input files based on the implemented empirical free energy function and the Lamarckian genetic algorithm. More specifically, the grid maps of the docking simulations were set with 60 grid points (with 0.375 Å spacing) in the x, y, and z directions centered on the benzene ring of the chemicals in active sites, which is a substrate-binding region, as demonstrated by the AutoGrid program. The parameters were as follows: trials = 200 dockings, population size = 150, random starting position and conformation, translation step range = 2.0 Å, rotation step range = 50°, maximum number of generations = 27,000, elitism = 1, crossover rate = 80%, local search rate = 6%, and 1.0 million energy evaluations. The docking results were sorted by the lowest binding energy of the most populated cluster in cases of convergence.

### 4.2. Plant Growth Conditions

Seeds of Napa cabbage (*Brassica campestris* L. ssp. *Pekinensis Rupr*.) and lentil beans (*Lens culinaris)* were purchased from ASIA seed company (Seoul, Korea), whereas rice seeds (*Oryza sativa*) were kindly provided by Prof. Min Chul Kim (Gyeongsang National University, Jinju, Korea). To conduct the seed germination and seedling growth upon sulfonamide contamination, seeds were sterilized with 3% NaClO and washed with sterilized distilled water five times. Sterilized seeds were placed onto 1.2% agar media containing 0 mg/L, 0.5 mg/L, or 5 mg/L of sulfonamide, i.e., sulfamethazine (SMZ), sulfathiazole (STZ), sulfadiazine (SDZ), or sulfamethoxazole (SMX), and grown vertically for seven days in a growth chamber (22 °C, 120 μE/m^2^/s illumination,) on a 16 h light/8 h dark cycle. Then, seed radicle emergence (i.e., rupturing of the seed coat), cotyledon opening (greening), and primary root growth were monitored. To measure the primary root length, photographs of captured *B. campestris* seedlings were measured using ImageJ (http://imagej.nih.gov/ij/dpwnload.html) (Bethesda, MD, USA) [80].

## 5. Conclusions

Although further uptake studies of sulfonamides from the soil to plants are required to understand the absorption and accumulation of veterinary medicines and their derived metabolites in plants, some of these studies [43,60] have suggested that the phyto-metabolism of antibiotics is a potentially significant route of human exposure to trace concentrations of antibiotics, which has prompted concerns about the development of antibiotic resistance in humans [76].

Furthermore, in figuring out the phytotoxic mechanisms of veterinary antibiotics, we now stand at the beginning stage. Here, we showed firstly in plants not only a molecular candidate of sulfonamide but also the quantification of relative inhibitory effects within four different sulfonamides: SMX, STZ, SDZ, SMZ. Some studies have demonstrated important variations in the phytotoxic effects of antibiotics on some plant species [35,39]; however, the relevant mechanisms by which most antibiotics influence plants remain poorly understood.

## Figures and Tables

**Table 1:** The summary of sulfonamides effects on plant.

Sulfonamide	Plant Species	Physiological Phenotype of Plants	Reference
**Sulfamethoxazole (SMX)**	*Cichaorium endivia,* *Cucumnus sativus*	seed germination	[34]
	*Oryza sativa*	seed germination and plant growth	[35]
	*Myriophyllum sibiricum,* *Lemma gibba*	plant growth and development	[36,37,38]
	*Daucus carota,* *Lactuca sativa*	root and shoot development, seed germination, and plant growth	[34,39,40]
	*Cucumis sativus,* *Arabidopsis thaliana,* *Ipomoea aquatica,* *Brassica rapa*	seed germination and growth inhibition	[35,41,42]
	*Medicago sativa*	root and shoot development	[39]
	*Lemna minor,* *Lemma gibba*	reduced plant growth	[36,37,38]
	*Lens culinaris,* *Oryza sativa,* *Brassica campestris*	seedling growth inhibition, primary root growth inhibition, and lateral root exposing	in this study
**Sulfathiazole (STZ)**	*Lactuca sativa*	plant growth	[43]
	*Lens culinaris,* *Oryza sativa,* *Brassica campestris*	seedling growth inhibition, primary root growth inhibition, and lateral root exposing	in this study
**Sulfadiazine (SDZ)**	*Triticum aestivum* *Cyphomandra betacea*	root and shoot elongation	[44,45]
	*Triticum aestivum,* *Apera spica-venti,* *Brassica napus*	plant growth and chlorophyll content	[46,47]
	*Salix fragilis,* *Zea mays,* *Corylus avellana,* *Arabidopsis thaliana*	plant growth and root alternation	[44,48,49,50]
	*Lens culinaris,* *Oryza sativa,* *Brassica campestris*	seedling growth inhibition, primary root growth inhibition, and lateral root exposing	in this study
**Sulfamethazine (SMZ)**	*Cichaorium endivia,* *Oryza sativa*	seed germination	[35]
	*Cucumnus sativus*	seed germination and plant growth	[34,35]
	*Phragmites autralis,* *Daucus carota,* *Lactuca sativa,* *Medicago sativa*	root growth and photosynthesis activity (hormetic response)	[39,40,51]
**Sulfamethazine (SMZ)**	*Medicago sativa*	root and shoot development	[39]
	*Lupinus luteus,* *Pisum sativum,* *Lens culinaris,* *Glycine max,* *Vigna angularis,* *Medicago sativa*	root decay and necrosis	[47,52]
	*Phragmites australis*	root development and leaf chlorophyll content	[53]
	*Hordeum vulgare*	root development	[54]
	*Lemma minor*	plant growth	[38]
	*Lens culinaris,* *Oryza sativa,* *Brassica campestris*	seedling growth inhibition, primary root growth inhibition, and lateral root exposing	in this study
**Sulfadimethoxine**	*Lythrum salicaria*	root growth and shoot development (hormetic response)	[55]
	*Amaranthus retroflexus, Plantago major,* *Remex acetosella*	root growth and shoot development	[56]
	*Cucumis sativus,* *Solanum ktcioersicum*	seedlings growth and development	[57]
	*Pamicum milliaceum,* *Pisum sativum,* *Zea mays*	root and stem growth inhibition, leave development, and biomass reduction	[47,58]
	*Hordeum vulgare*	root hair and root growth, root development, and photosynthetic pigment	[47,59]
	*Salix fragilis*	root morphology	[41,60]
	*Lactuca sativa,* *Medicago sativa*	root growth	[39]
**Sulfamethoxine**	*Amaranthus retroflexus*	plant growth and development, post-germination	[40]
	*Cucumis sativus*	seed germination and growth inhibition	[45]
	*Panicum miliaceum,* *Brassica rapa,* *Ipomoea aquatica*	plant growth and development	[42,58]
	*Panicum miliaceum,* *Plantago major,* *Zea mays,* *Hordeum disthicum,* *Rumex acetosella,* *Pisum sativum*	plant growth and development	[40,47,55]
